# Cotylenin A inhibits cell proliferation and induces apoptosis and *PAX6* mRNA transcripts in retinoblastoma cell lines

**Published:** 2010-06-04

**Authors:** Yoshiko Kashiwagi, Nobuo Kato, Takeshi Sassa, Koichi Nishitsuka, Teiko Yamamoto, Hiroshi Takamura, Hidetoshi Yamashita

**Affiliations:** 1 Department of Ocular Cellular Engineering, Faculty of Medicine, Yamagata University, Yamagata City, Yamagata, Japan; 2 The Institute of Scientific and Industrial Research, Osaka University, Ibaraki City, Osaka, Japan; 3 Department of Bioresource Engineering, Faculty of Agriculture, Yamagata University, Tsuruoka City, Yamagata, Japan; 4 Department of Ophthalmology and Visual Sciences, Faculty of Medicine, Yamagata University, Yamagata City, Yamagata, Japan

## Abstract

**Purpose:**

Retinoblastoma, a childhood cancer of the retina, is caused by inactivation of the tumor suppressor gene retinoblastoma (*RB*). Cotylenin A (CN-A), a novel fusicoccane-diterpene glycoside, accelerates the differentiation of several types of myeloid cell lines and is a candidate for a new type of anticancer therapeutic agent with this effect. However, whether CN-A has the same effect on retinoblastoma cells is unknown. We studied the response of two retinoblastoma cell lines, Y-79 and WERI-Rb-1, to CN-A.

**Methods:**

We studied the response of two retinoblastoma cell lines to CN-A with respect to cell growth, apoptosis, morphology, mRNA, protein expression analysis of specific genes (*N-myc*, cyclin-dependent kinase inhibitor 1A [*P21*], paired box gene 6 [*PAX6*], and rhodopsin [*RHO*]), and activity of three PAX6 promoters (P0, P1, and Pα).

**Results:**

CN-A inhibited cell proliferation and induced apoptosis via caspase activity in the two retinoblastoma cell lines. In addition, CN-A induced mRNA expression of *P21, PAX6,* and *RHO* and protein expression of P21. In Y-79 cells, *PAX6* P1 promoter was activated by CN-A. In WERI-Rb-1 cells, *PAX6* P0, P1, and Pα promoter were activated by CN-A. CN-A decreased mRNA and protein expression of *N-myc* in two retinoblastoma cell lines.

**Conclusions:**

The responses of retinoblastoma cells to CN-A include inhibition of cell growth, induction of apoptosis, and the potential to change neuroblastoma characteristics of retinoblastoma cells.

## Introduction

Retinoblastoma, a childhood cancer of the retina [1], is caused by an inheritable mutation of the tumor suppressor gene retinoblastoma (*RB*) [2]. Retinoblastoma has bipotential differentiation status and Müller- and photoreceptor-like characters [3]. Recently, the possibility that retinoblastoma results from a mixture of transformed retinal progenitor and transition cells was suggested [4]. Although the inactivation of the two *RB* alleles is the most important event in the oncogenesis of retinoblastoma, other oncogenes or tumor suppressor genes may also be involved in the aggressive progression of this tumor. Various therapies (e.g., chemotherapy, cryotherapy, thermotherapy, and radiation therapy) have been used to cure retinoblastoma [5].

Cotylenin A (CN-A), a novel fusicoccane-diterpene glycoside isolated from the culture filtrate of a fungus (*Cladosporium* sp.), is a plant growth regulator with cytokinin-like activity [6–8]. CN-A, which also has the ability to induce differentiation in several human and murine myeloid leukemia cell lines [9,10], significantly stimulated both functional and morphologic differentiation of leukemic cells in nine of 12 cases [11]. It has been reported that the cptylenin A- induced differentiation of human leukemia cell lines is independent of the transforming growth factor-beta signaling system [12]. Combined treatment with interferon-α (IFN-α) and CN-A has induced apoptosis of human lung cancer cells [13]. In addition, this treatment has significantly inhibited the growth of both xenografted lung cancer cells, without apparent adverse side effects [13], and primary ovarian carcinoma cells [14]. Combined treatment with rapamycin and CN-A inhibited cell growth in breast carcinoma in vitro and in vivo [15]. Thus, CN-A is among the unique agents that accelerate cell differentiation [16]. Recently, CN-A was reported to be a molecule that binds to a 14–3–3 regulator protein complex [17]. However, whether CN-A has this effect on retinoblastoma cells is not known.

In this study, we examined the effect of CN-A on the proliferation and differentiation of human retinoblastoma cell lines Y-79 and WERI-Rb-1 (WERI) [18,19] to investigate whether retinoblastoma cells also respond to CN-A.

## Methods

### Cell culture

Retinoblastoma cell lines Y-79 and WERI were obtained from the American Type Culture Collection (Manassas, VA). Y-79 and WERI cells were cultured on Roswell Park Memorial Institute (RPMI) 1640 medium (Invitrogen, Carlsbad, CA) containing 10% fetal bovine serum (JRA Bioscience, Lenexa, KS). Cells were grown in a humidified 5% CO_2_ atmosphere at 37 °C.

### Cotylenin A

CN-A was purified from the ethyl acetate extract of the culture filtrate of *Cladosporium* 501–7W [7,8]. CN-A was dissolved in dimethyl sulfoxide at a 20-mg/ml concentration and then added to the medium at concentrations of 0, 10, or 20 μg/ml. Dimethyl sulfoxide at concentrations up to 0.1% had no effect on cell proliferation, gene expression, or morphology when added with or without CN-A.

### Assay of cell growth

Cell Titer-Blue Assay (Promega, Madison, WI) was used to evaluate cell growth. Cells (1.0×10^5^ cells/ml) were cultured with 0, 10, or 20 μg/ml CN-A for 0, 3, and 7 days in 96-well plates (CELLSTAR^®^; Greiner Bio-One, Frickenhausen, Germany). Then, 20 μl of the Cell Titer-Blue Assay solution was added, and the cells were incubated for 2 h at 37 °C and 5% CO_2_. Fluorescence (560/590 nm) was measured using a Gemini EM microplate spectrofluorometer (Molecular Devices, Sunnyvale, CA). The Mann–Whitney *U*-test (n=3) was used for statistical analysis.

### Terminal deoxyribonucleotidyl transferase (TdT) -mediated deoxyuridine 5′-triphosphate -biotin nick-end labeling assay (TUNEL) assay

Cells (1.0×10^5^ cells/ml) were incubated with or without 10 μg/ml CN-A for 7 days in six-well plates (CELLSTAR^®^). At the end of the treatment, the cells were washed with PBS (137 mM NaCl, 2.7 mM KCl, 10 mM Na_2_HPO_4_/KH_2_PO_4_, pH 7.4.) and centrifuged for 5 min at 500× g for microscope slide cell preparations. We used the Apoptosis In Situ Detection Kit (Wako Pure Chemical Industries, Osaka, Japan) to evaluate cell apoptosis. Cells were fixed with 4% formalin neutral-buffered solution for 10 min. The 3′ terminals of DNA fragments, which in TUNEL positive cells was labeled by TdT protein, Peroxidase-conjugated TdT antibody and 3,3′-diaminobenzidine (DAB). The samples were covered with Soft Mounting Media (Wako Pure Chemical Industries) and observed with a laser scanning microscope (LSM-510 Meta; Carl Zeiss MicroImaging, Thornwood, NY). Under the microscope, the total number of cells in 25 random fields per condition was counted: incidence=terminal transferase uridyl nick end labeling (TUNEL)-positive cells/all cells. The Mann–Whitney *U*-test (n=3) was used for statistical analysis.

### Observation of morphological changes and cell counting

For morphological examination, cells were cultured in poly-D-lysine-coated tissue culture vessels (CELLSTAR^®^). Cells were examined and photographed using a Leica DMI3000B inverted microscope (Leica, Wetzlar, Germany). Under the microscope, the total number of cells in ten random fields per condition was counted: dendrite-like process cells/all cells. The Mann–Whitney *U*-test (n=3) was used for statistical analysis.

### Reverse-transcriptase PCR analysis

Total RNA was prepared by disrupting the cells in ISOGEN reagent (Nippon Gene, Toyama, Japan), and isopropanol precipitation. Total RNA (2 μg) was reverse transcribed with 200 U reverse transcriptase (Promega), 0.5 μg oligo (dT)_16_ primer, and 20 U RNase inhibitor (Takara Bio, Shiga, Japan) for 60 min. PCR amplifications were performed in 25-μl reaction mixtures containing 1.5 μl of the resulting cDNA, 200 μM deoxycytidine triphosphate (adenine, cytosine, guanine, and thymine) mixture (dNTP), 1 μM primers, and 1 U KOD® plus (Toyobo, Osaka, Japan). The resulting cDNAs were amplified using a KOD-Plus-PCR Kit (Toyobo) in 25 µl of medium. Primer sequences are detailed in Table 1. The PCR schedule was as follows: 1 min at 95 °C, followed by 20 (β-actin; *ACTB*) or 25–35 cycles, depending on the primer (other experiments); 15 s at 94 °C, 30 s at 60 °C or 55–65 °C, depending on the primer (other experiments); and a final extension step of 30 s at 68 °C and 1.5 min at 68 °C. Annealing temperatures and the number of PCR cycles are detailed in Table 1.

**Table 1:** PCR primers and PCR schedules.

**Gene**	**Nucleotide (5′-3′)**	**Accession number**	**Product (bp)**	**Anneaing (°C)/cycles**
*β-Actin*	F: CCCATGCCATCCTGCGTCTG	NM_001101	573	60 °C/20 cycles
	R: CGTCATACTCCTGCTTGCTG			
*PAX6*	F: ATGGTTTTCTAATCGAAGGG	NM_000280	149	58 °C/33 cycles
	R: CGGTGTGGTGGGTTGTGGAAT			
*RHO*	F: CATCGAGCGGTACGTGGTGGTGTG	NM_000539	577	65 °C/33 cycles
	R: GCCGCAGCAGATGGTGGTGAGC			
*P21*	F: CTCCAAGAGGAAGCCCTAATCC	NM_000389	535	60 °C/25 cycles
	R: TTTGATGATGCCCCCACTCG			
*N-myc*	F: GACCACAAGGCCCTCAGTAC	NM_005378	240	60 °C/25 cycles
	R: GTGGATGGGAAGGCATCGTT			
*PAX6-P0 *promoter	F: GGTACCTTTTCCTATGAGGGCAAGAC	NG_008679	5315	60 °C/30 cycles
	R: GCTAGCTCCCTCAGTAACTCGCTTCC			
*PAX6-P1 *promoter	F:GGTACCCTGCAAAAAGAGAGACGTTTGGGGC	NG_008679	3796	60 °C/30 cycles
	R: GCTAGCCTTTATGAGGCATCCTTTCTGG			
*PAX6-Pa *promoter	F: GGTACCCAGAGCCAGCATGCAGAACA	NG_008679	3628	60 °C/30 cycles
	R: GCTAGCCTGATTCACTCCGCTGTGAC			
*PAX6* (exon 11–13)	F: AACAGACACAGCCCTCACAAACA	NM_000280	275	55 °C/35 cycles
	R: CGGGAACTTGAACTGGAACTGAC			
*PAX6* (exon 3–6)	F: GGAAGACTTTAACTAGGGGC	NM_000280	416	58 °C/33 cycles
	R: ATGGACGGGCACTCCCGCTT			

The PCR products were separated using 2% agarose gel (Iwai Chemicals, Tokyo, Japan) electrophoresis and visualized via ethidium bromide staining. Results were quantified using CS Analyzer software (ATTO Corporation, Tokyo, Japan).

### Western blot

Cells (1.0×10^5^ cells/ml) were incubated with or without 10 μg/ml CN-A for 5 days in six-well plates (CELLSTAR^®^). Cells were collected and lysed in sample radio immunoprecipitation assay (RIPA) buffer (50 mM Tris-HCl [pH 8.0], 150 mM NaCl, 1 mM EDTA, 1% Triton X-100, 0.1% sodium dodecyl sulfate [SDS], 0.1% sodium deoxycholate) with an added protease inhibitor cocktail (Roche Diagnostics, Basel, Switzerland) and 1 mM phenylmethylsulphonyl fluoride (Wako Pure Chemical Industries) at 4 °C. After sonication in ice water, crude lysates were cleared via centrifugation at 22,000× g for 30 min at 4 °C. The total protein concentration of the lysates was measured using the Bradford assay. Lysate aliquots were diluted at a 3:1 ratio with sample buffer (50 mM Tris-HCl [pH 6.8], 10% weight/volume [w/v] SDS, 10% v/v glycerol, 10% v/v 2-mercaptoethanol, 0.02% w/v bromophenol blue) and boiled 2 min. Equal amounts of protein (40–50 μg) were loaded onto 10% and 12.5% SDS-polyacrylamide gels and subjected to electrophoresis. The separated proteins were then electrotransferred to polyvinylidene fluoride (PVDF) membranes (Immun-Blot^®^ PVDF membrane; Bio-Rad, Hercules, CA). Following electrotransfer, the blots were incubated for 60 min in a blocking solution (0.3% w/v dried low-fat milk/Tris-buffered saline) on an orbital shaker. The primary antibodies used in the blocking solution were anti-rhodopsin (antimouse; Sigma-Aldrich, St. Louis, MO); 1 μg/ml anti-*PAX6* (antirabbit #PRB-278P; Covance, Princeton, NJ); anti-N-myc diluted 1:500 (antirabbit #9405; Cell Signaling Technology, Beverly, MA); anti-cleaved-poly (ADP-ribose) polymerase (PARP) diluted 1:500 (antirabbit #9541; Cell Signaling Technology); 1 μg/ml anti-cyclin-dependent kinase inhibitor 1A (P21, #556430; BD Biosciences, San Jose, CA); anti-acetyl histone H3 diluted 1:1,000 (antirabbit #9671; Cell Signaling Technology); anti-acetyl histone H4 diluted 1:1,000 (antirabbit #2594; Cell Signaling Technology); 20 ng/ml anti-β-actin (antimouse #A5316; Sigma-Aldrich), and 1 μg/ml secondary horseradish peroxidase conjugate (mouse and rabbit; GE Healthcare, Little Chalfont, UK). The ECL™ Plus Western Blotting Detection System (GE Healthcare) was used for detection. Results were quantified using CS Analyzer software. The Mann–Whitney *U*-test (n=3) was used for statistical analysis.

### Promoter assay

The pGL4.10-promoter vector was constructed using designed primers (Table 1) and the restriction enzymes KpnI and Nhe I. Cells were seeded in duplicate into 24-well plates (CELLSTAR^®^) at a density of 1×10^5^ cells/well with growth medium. After 24 h, cells were transfected for 16 h with pGL4.10-promoter constructs and pGL4.74 plasmid (Promega) as an internal control for transfection efficiency using FuGENE HD (Roche Diagnostics). Cells were treated with or without 10 μg/ml CN-A for 48 h. Luciferase activity in cells was measured using the Dual-Luciferase Reporter Assay System (Promega) with luminometer LB9507 (Berthold Technologies, Bad Wildbad, Germany). The Mann–Whitney *U*-test (n=3) was used for statistical analysis.

### RNA interference experiment

A human *PAX6*-specific double-stranded small interfering (si)RNA was synthesized (Silencer Select Pre-Designed siRNA ID#s10067; Ambion, Austin, TX). A lipid transfection system (siPORT NeoFX transfection agent; Ambion) was used to introduce siRNAs into Y-79 and WERI cells via the following protocol: cells were preincubated with or without 10 μg/ml CN-A for 24 h, after which they were resuspended at a density of 10^6^ cells/ml growth medium. One milliliter was seeded in duplicate into 24 wells (CELLSTAR^®^). The mixtures of siRNA and lipid reagent were added to 10^6^ cells at a final concentration of 100 nM. Thereafter, cells were treated with or without 10 μg/ml CN-A for 48 h. Reverse transcriptase (RT)–PCR was conducted as described above.

## Results

### Cotylenin A inhibition of cell growth in two retinoblastoma cell lines

**Figure 1:**
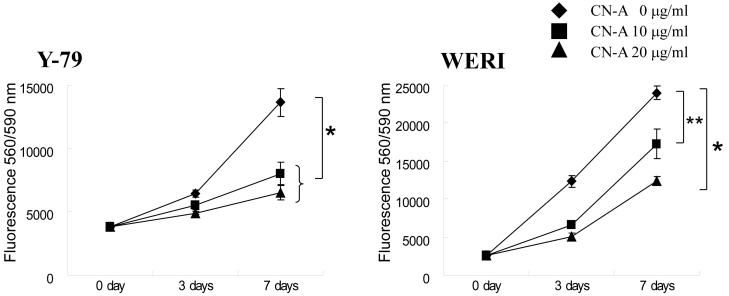
Cell growth response in retinoblastoma cell lines. The response of cotylenin A (CN-A) on retinoblastoma cell growth was examined using fluorescence cell viability assays repeated in triplicate. Retinoblastoma cells were treated with CN-A at concentrations of 0, 10, or 20 μg/ml and incubated for 0, 3, and 7 days (n=3); the error bar indicates the standard deviation. Asterisks indicate significant differences when compared with the control without CN-A (*p<0.01; **p<0.05).

To determine if CN-A inhibits cell growth, we investigated the cell viability of two retinoblastoma cell lines, Y-79 and WERI, in growth medium with 0, 10, and 20 μg/ml CN-A for 0, 3, and 7 days (Figure 1). At 10 and 20 μg/ml, CN-A significantly repressed the proliferation compared to 0 μg/ml in two cell lines. At 10 and 20 μg/ml CN-A for 3 and 7 days, Y-79 and WERI cell growth increased compared to control.

### Cotylenin A induction of apoptosis in two retinoblastoma cell lines

**Figure 2:**
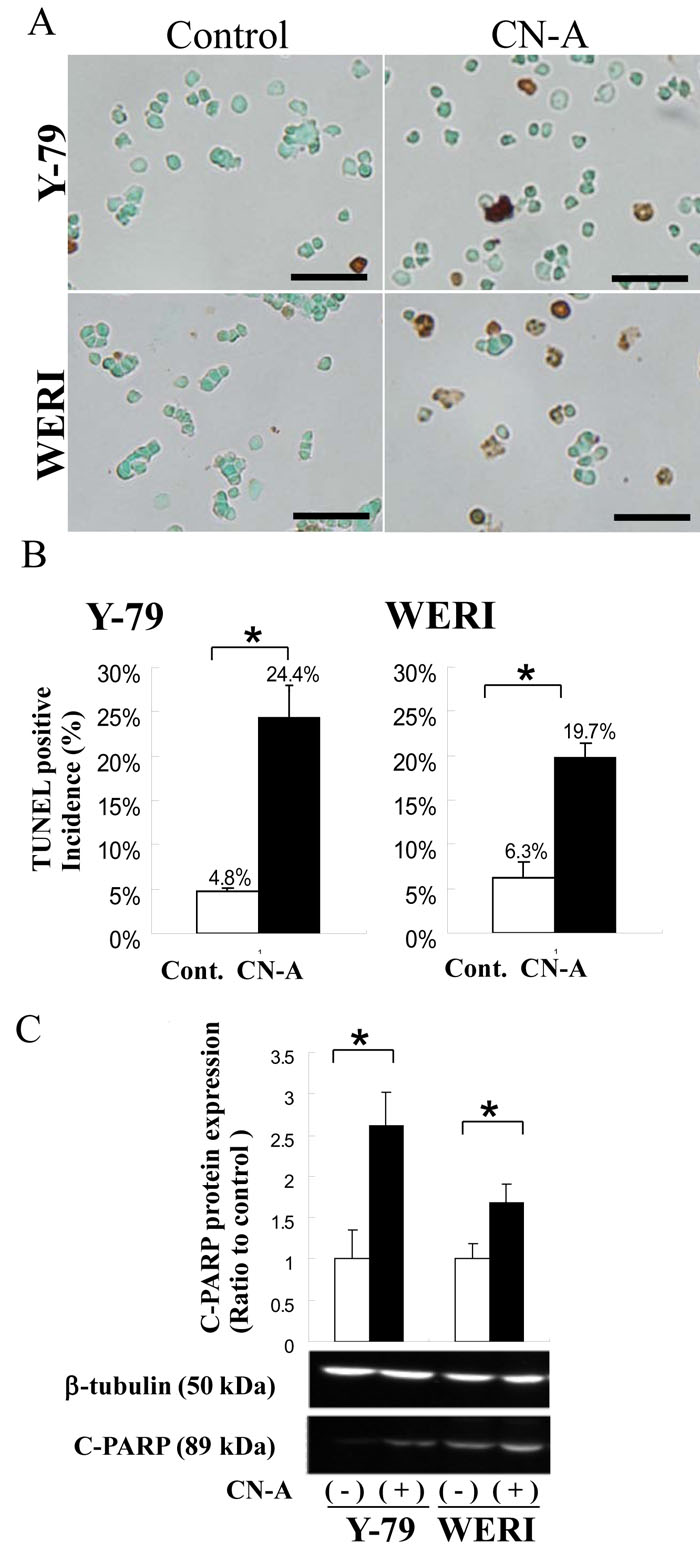
Response of cotylenin A on cell death and apoptosis in retinoblastoma cell lines. **A**: Detection of apoptosis using a terminal deoxynucleotidyl transferase deoxyuridine 5′-triphosphate (dUTP) nick-end labeling (TUNEL) assay. Cells were treated with or without 10 μg/ml cotylenin A (CN-A) for 7 days. The bar represents 25 μm. The arrow points to TUNEL-positive cells. **B**: The bar graph indicates the percentage of TUNEL-positive cells. Asterisks indicate significant differences when compared with the control without CN-A (p<0.05; **C**) the bar graph and electrophoresis photographs indicate the protein expression of cleaved- Poly Adenosine diphosphate –ribose polymerase (PARP) in retinoblastoma cells. The bar graph indicates C-PARP protein expression (n=3); the error bar indicates the standard deviation. Asterisks indicate significant differences when compared with the control without CN-A (p<0.05).

CN-A has been reported to induce apoptosis in human lung carcinoma cells [13]. To investigate whether CN-A also induces apoptosis in a retinoblastoma cell, we examined retinoblastoma cell lines cultured with or without 10 μg/ml CN-A for 7 days in noncoated tissue culture vessels using a TUNEL assay (Figure 2A,B) and western blot analysis (Figure 2C). CN-A treatment increased the number of TUNEL-positive cells threefold compared to untreated cells in the two cell lines (Figure 2B). Cleaved PARP can be used as a marker of the early stages of apoptosis because the PARP protein is cleaved by caspase-3 when apoptosis is induced [20,21], with nuclear condensation and fragmentation of chromosomal DNA occurring in the final stage of apoptosis [22]. Cleaved-PARP proteins in two retinoblastoma cell lines accumulated (Figure 2C).

### Cotylenin A-induced morphological change in two retinoblastoma cell lines

**Figure 3:**
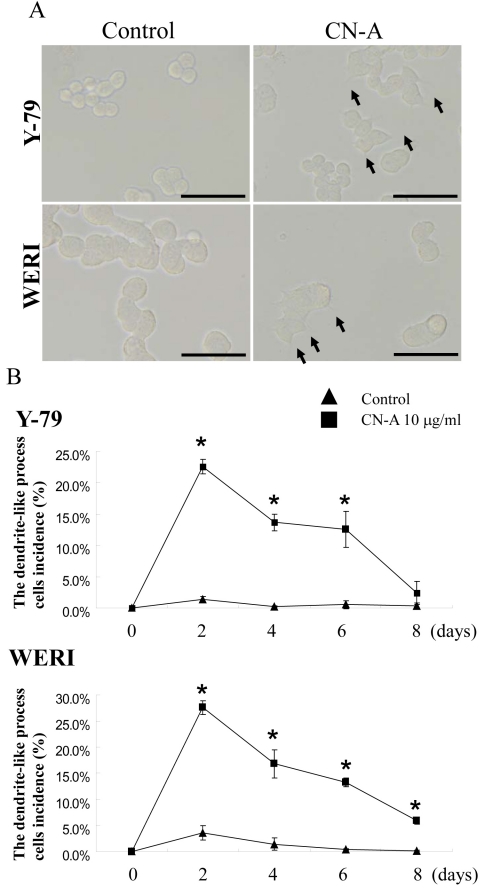
Photographs of the morphology of retinoblastoma cell lines with and without cotylenin A. **A**: Photographs indicate morphology of retinoblastoma cells with or without 10 μg/ml CN-A for 2 days. The bars indicate the length of 25 μm. **B**: The graph indicates the percentage of the dendrite-like process cells (mean±SD, n=3). Asterisks indicate significant differences when compared with the control without CN-A (*p<0.05).

To investigate whether CN-A caused morphological changes to retinoblastoma cells, we observed two retinoblastoma cell lines cultured with 10 μg/ml CN-A for 7 days in poly-d-lysine-coated tissue culture vessels (Figure 3) to allow adherence of the retinoblastoma cells, which are normally cultured in suspension. CN-A induced morphological changes in Y-79 and WERI cells, including the appearance of dendrite-like processes (Figure 3A, right). Figure 3B shows the percentage of the dendrite-like process cells at 0, 2, 4, 6, and 8 days with CN-A treatment. The percentage of the morphologically changed cells increased significantly by CN-A. At 2 days, the percentage was maximal for both Y-79 and WERI cells. The percentage of morphologically changed cells gradually declined at 4, 6, and 8 days.

### Cotylenin A induced and decreased gene expression

**Figure 4:**
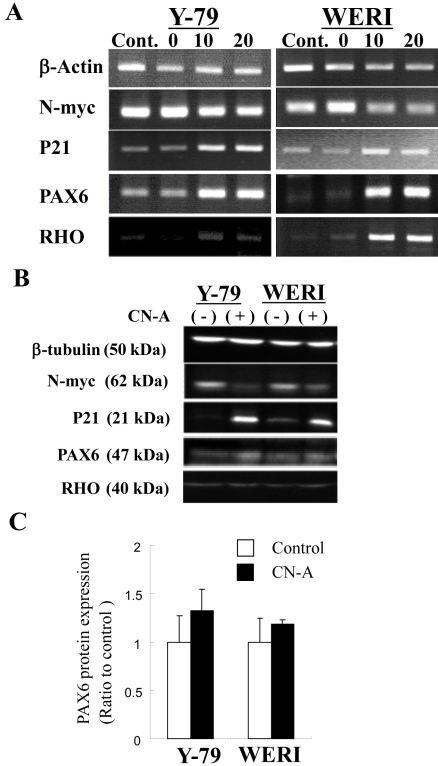
Expression of mRNA and protein in retinoblastoma cells. **A**: Some gene mRNA expression was investigated using reverse transcription polymerase chain reaction (RT–PCR). Cells were treated with or without 10 μg/ml cotylenin A (CN-A) for 3 days. The numbers (0, 10, and 20) indicate CN-A concentrations (μg/ml). *β-Actin* mRNA was used as an internal control. **B**: Some protein expression was investigated using western blot analysis in retinoblastoma cells. Cells were treated with or without 10 μg/ml CN-A for 5 days. “Cont.” indicates the non-treatment. “CN-A” indicates the CN-A treatment (10 μg/ml). β-Tubulin protein was used as a loading control. **C**: The bar graph indicates the PAX6 protein expression (n=3); the error bar indicates the standard deviation.

We postulated that CN-A induced mRNA and protein expression of some genes in two retinoblastoma cell lines (Figure 4). *N-myc* has been reported to be expressed in retinoblastoma tumors and fetal retinas (but not adult retinas) [23] and to decrease during differentiation of neuroblastoma [24] and retinoblastoma cell lines [25]. In Y-79 and WERI cells, CN-A reduced *N-myc* mRNA and protein expression. *P21* has been reported to regulate cell-cycle progression at G_1_ [26]. In Y-79 and WERI cells, CN-A induced *P21* mRNA and protein expression. *PAX6*, a highly conserved transcription factor in vertebrates, is crucial for the development of the central nervous system, eye, nose, pancreas, and pituitary gland (reviewed in [27]). In Y-79 and WERI cells, CN-A induced *PAX6* mRNA expression. In two cell lines, few increases of PAX6 protein expression were detected; however, significant increases of PAX6 protein were not detected at p<0.05 (Figure 4C). *RHO* is a rod photoreceptor marker gene [28]. *RHO* expression increased in CN-A-treated Y-79 and WERI cells compared to untreated cells. In two cell lines, alternation of RHO protein expression was not detected.

### Cotylenin A-induced *PAX6* promoter activity in two cell lines

**Figure 5:**
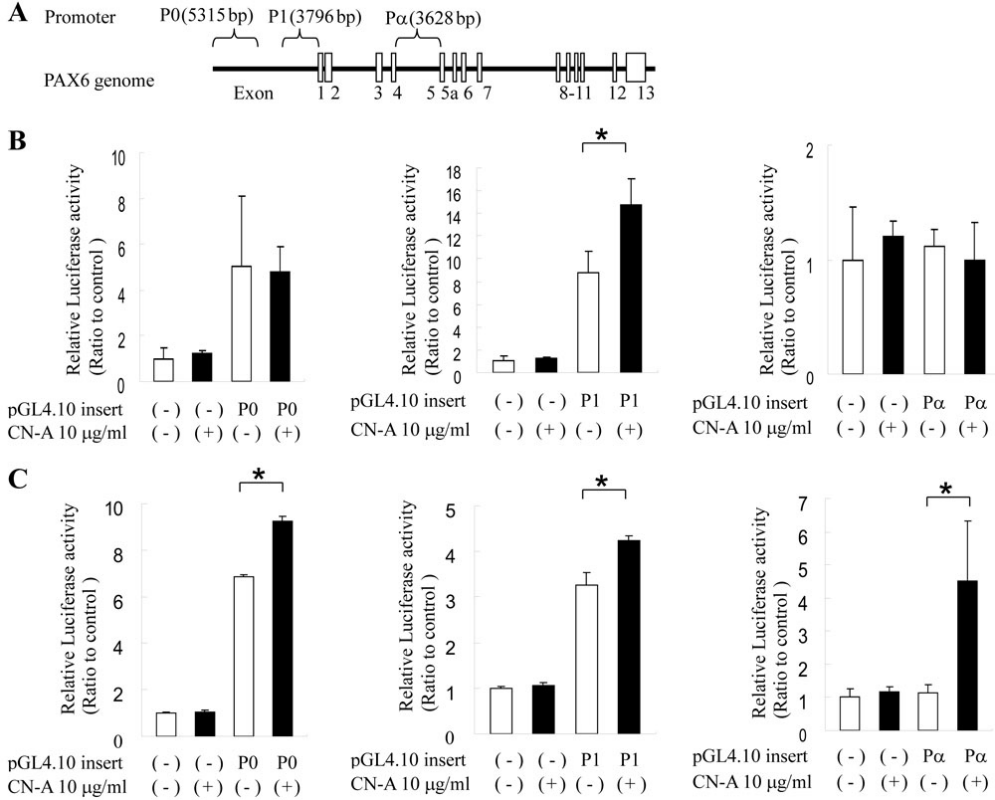
Luciferase assay of paired box gene 6 (PAX6) promoter activity. **A**: Schematic indicates the representation of the PAX6 transcript unit in humans. The boxes denote exons. Braces indicate the extent of P0, P1, and Pα promoters in the *PAX6* gene area. **B**: Luciferase assay of P0, P1, and Pα promoter activity in Y-79 cells. **C**: Luciferase assay of P0, P1, and Pα promoter activity in WERI cells. P0 of pGL4.10 insert indicates the insertion of P0 promoter sequences (5315 bp), P1 promoter activity (3796 bp), and Pα promoter sequences (3628 bp). White and black squares indicate the no-CN-A treatment and CN-A treatment, respectively. The negative pGL4.10 insert indicates an empty sequence. The data are presented after normalizing transfection efficiency using the *Renilla* luciferase reporter gene (n=3); the error bar indicates the standard deviation; Mann–Whitney *U*-test, *p<0.05.

Three promoters of *PAX6* (P0, P1, and Pα) have been identified (reviewed in [29]; Figure 5A). To confirm whether CN-A activates the *PAX6* promoter, we investigated the luciferase activity of the promoters in two retinoblastoma cell lines after 48 h treatment with 10 μg/ml CN-A. In Y-79 and WERI cells, the P0 and P1 promoters were activated without CN-A treatment. With CN-A treatment the luciferase activity of only the *PAX6* P1 promoters increased compared to that of the untreated control in Y-79 (Figure 5B). In WERI cells the luciferase activity of the three promoters with CN-A increased compared to that of the untreated control (Figure 5C).

### Cotylenin A-induced Δexon5 *PAX6* mRNA in WERI cells

**Figure 6:**
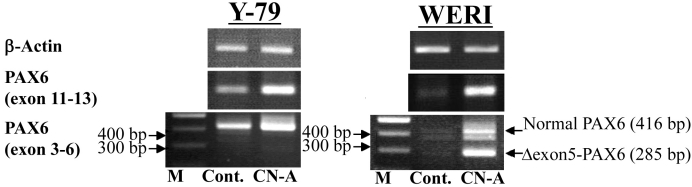
The mRNA expression of paired box gene 6 (*PAX6)* spliced variants in retinoblastoma cell lines. Cells were treated with or without 10 μg/ml CN-A for 3 days. “Cont.” indicates the non-treatment. “CN-A” indicates the CN-A treatment (10 μg/ml). *β-Actin* mRNA was used as an internal control.

The Pα promoter transcript has been reported to encode paired domain-less (ΔPD) *PAX6* [30,31]. To confirm whether CN-A treatment induced the expression of spliced *PAX6* variants, we used RT–PCR with various primer sets to investigate transcription products. The *PAX6* (exons 11–13) primer set gave results similar to that shown in Figure 4A. The shift-down *PAX6* (exons 3–6) mRNA band in WERI cells with CN-A treatment was detected (Figure 6). This shift-down mRNA band sequencing revealed the loss of exon 5 (position 544–674 in NM_000280). We termed these spliced variants Δexon5 *PAX6* mRNA. However, in Y-79 cells, Δexon5 *PAX6* mRNA expression was not detected.

### Increase and decrease in the pattern of cotylenin A-induced mRNA expression of *PAX6* and *RHO*

**Figure 7:**
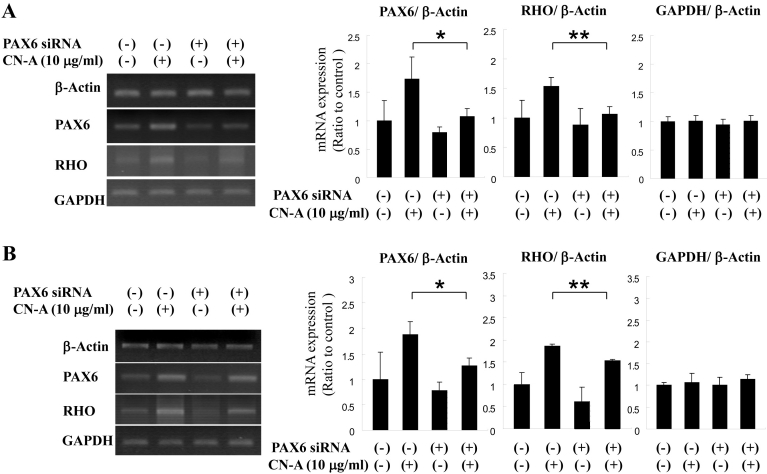
Response of paired box gene 6 (PAX6) knockdown on rhodopsin (*RHO)* expression inretinoblastoma cell lines. *PAX6* siRNA was introduced into Y-79 and WERI cells via siPORT for knockdown of *PAX6* mRNA. The electrophoresis photographs and the bar graphs indicate the result of reverse transcription polymerase chain reaction (RT–PCR) in Y-79 cells (**A**) and WERI cells (**B**). *PAX6* and *RHO* mRNA expression were investigated using RT–PCR. The mRNA expression was detected 48 h after siRNA transfection and cotylenin A (CN-A) treatment. The bar graph indicates transduction of *PAX6*, *RHO*, and *GAPDH* mRNA expression by *PAX6* siRNA (n=3); the error bar indicates the standard deviation; Mann–Whitney *U*-test, *p<0.05, **p<0.01.

To further study the regulatory effect of *PAX6* on *RHO* expression, we conducted *PAX6* RNA interference experiments (RNAi). Endogenous *PAX6* mRNA was knocked down by the RNAi technique to verify a regulatory correlation between *PAX6* and *RHO* expression. RT–PCR showed that *PAX6* siRNA significantly knocked down *PAX6* mRNA expression in transfected cells with CN-A treatment in both Y-79 and WERI cells (Figure 7). *RHO* mRNA expression levels with *PAX6* siRNA and CN-A decreased and were similar to *PAX6* mRNA expression levels in both Y-79 and WERI cells. In contrast, glyceraldehyde-3-phosphate dehydrogenase mRNA expression showed no change. The mRNA expression level of *PAX6* and *RHO* in retinoblastoma cells with PAX6 siRNA and without CN-A decreased compared to control; however, this expression level showed no significant difference at p<0.05.

### Cotylenin A did not have the ability of a histone deacetylase inhibitor

**Figure 8:**
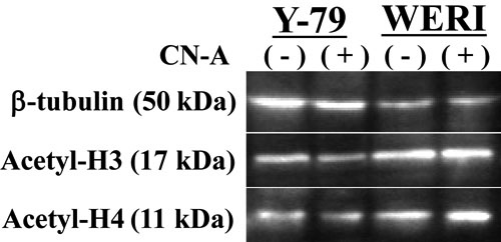
The accumulation of acetylated histone H3 and H4 protein in retinoblastoma cell lines. Western blot analysis of acetylated histone H3 and H4 (acetyl-H3 and H4) protein levels in retinoblastoma cells treated with (+) or without (–) cotylenin A (CN-A) for 5 days. β-Actin was used as a loading control.

Some differentiation agents have been known to exert histone deacetylase inhibitor activity (reviewed in [32]). In retinoblastoma cell lines, sodium butyrate has been reported to be the histone deacetylase inhibitor agent to have the ability of differentiation agents [33,34]. Therefore, we next examined cellular levels of acetylated histone H3 and H4 in the presence or absence of CN-A treatment. Western blot analysis showed that the accumulation of acetylated H3 and H4 in Y-79 and WERI cells were unchanged by CN-A treatment (Figure 8).

## Discussion

We showed that CN-A inhibits cell proliferation and induces cell death in two retinoblastoma cell lines (Figure 1 and Figure 2) as it does in other tumor cell lines [13–15,35]. Cleaved PARP western blot analysis results (Figure 2C) suggest that CN-A-induced apoptosis of retinoblastoma cells is caspase mediated. These results indicated that the response of retinoblastoma cells to CN-A is similar to that of other tumor cells [13,16].

CN-A induced Y-79 and WERI cells to exhibit dendrite-like processes (Figure 3, right), an effect similar to that of other differentiation reagents [25,33,36]. CN-A may dynamically change cell adhesion and cytoskeleton conformation of the retinoblastoma cell lines Y-79 and WERI cells. The elongation of CN-A induced-dendrite-like processes was not detected with continuous observation. In addition, the number of dendrite-like process cells gradually declined. With continuous observations, morphologically changed cells were found to die. Therefore, the morphological change by CN-A may be related to CN-A-induced apoptosis.

CN-A reduced the expression of the oncogene *N-myc*, a neuroblastoma marker that has been reported to be expressed in retinoblastoma tumors and fetal retinas (but not adult retinas) [23], and to decrease during the differentiation of neuroblastoma [24] and retinoblastoma cell lines [25]. This suggests that the neurotumor state of retinoblastoma cell lines changes by CN-A.

CN-A increased the mRNA and protein expression of *P21* in Y-79 and WERI cells (Figure 4A,B). P21 protein has been reported to regulate cell-cycle progression at G1 [26]. In Y-79 and WERI cells, CN-A is considered to induce not only apoptosis but also cell-cycle inhibition.

CN-A increased the mRNA expression of *PAX6* in Y79 and WERI cells (Figure 4A). *PAX6* has been reported to play multiple roles in both lens and retinal development [37] and is required for the proliferation and expansion of retinal stem cells [38]. Three promoters of *PAX6* (P0, P1, and Pα) have been identified (reviewed in [29]; Figure 5A). In addition, PAX6 has three isoforms: normal PAX6, PAX6 (5a), and paired domain-less (ΔPD)PAX6. The P0 and P1 promoter transcripts have been reported to encode normal PAX6 and PAX6 (5a) splice variants. It has been reported that (ΔPD)*PAX6* mRNA is transcribed by activation of the Pα promoter [30,31]. CN-A-induced *PAX6* splice variant mRNA expression patterns also differed between the two retinoblastoma cell lines, corresponding to activation of three *PAX6* promoters by CN-A (Figure 6). Normal *PAX6* mRNA translated into a 47-kDa protein with 422 amino acids. The mass of the deduced amino acid of Δexon5 *PAX6* mRNA was about 32 kDa and contained 286 amino acids. It is presumed that the deduced amino acid of Δexon5 PAX6 is (ΔPD)PAX6 proteins [29]. Δexon5 *PAX6* mRNA expression in WERI was considered to be transcribed by *PAX6* Pα promoter activity. A western blot investigation on the expression of the Δexon5 PAX6 protein induced by CN-A in WERI cells showed that the mass of the deduced amino acid of Δexon5 *PAX6* mRNA was not detected with or without CN-A-treatment (data not shown). Overexpression of (ΔPD)-*PAX6* causes a severe microphthalmic phenotype due to apoptotic cell death in the lens during embryonic development [31,37]. Little is known about the molecular mechanism and function of (ΔPD)-*PAX6*. We attempted to overexpress the Δexon5 *PAX6* mRNA in WERI cells; however, *RHO* gene expression change was not detected (data not shown). The (ΔPD)-*PAX6* mRNA expression in WERI cells by CN-A may be related to apoptosis or another cell physiology. The different response to CN-A on *PAX6* transcription in two retinoblastoma cell lines is unique. CN-A may be useful in researching the expression of *PAX6* splice variants.

*RHO* is a rod photoreceptor marker gene [28] that is modulated by *PAX6* in *Drosophila* [39]. In Y-79 and WERI cells, CN-A induced *RHO* mRNA expression (Figure 4A) but it did not induce RHO protein expression (Figure 4B). In the *PAX6* siRNA experiment in Y-79 and WERI cells with CN-A treatment, the expression pattern of *PAX6* mRNA was similar to that of *RHO* mRNA (Figure 7). These results indicate that *PAX6* mRNA expression may participate in the expression of *RHO*. CN-A-induced *PAX6* is considered to modulate the expression of *RHO* mRNA. In addition, CN-A may induce rod photoreceptor differentiation in retinoblastoma cell lines.

The reagents that promote the differentiation of retinoblastoma cell lines include retinoic acid (RA)-induced cone photoreceptor-specific genes in WERI cells [35]. RA has been used in combination with other reagents to induce photoreceptor differentiation of embryonic stem cells [40]. Butyrate has been reported to induced *Recoverin*, the rod photoreceptor marker gene, mRNA, and protein in Y-79 cells [41] and dibutyryl cyclic adenosine 3′, 5′-monophosphate (AMP) and butyrate-induced morphological changes in Y-79 cells [25,33]. In particular, butyrate has been reported to be a histone deacetylase inhibitor agent [32,33]. However, CN-A does not have the ability to inhibit histone deacetylase. Treatment with a combination of CN-A and vitamin D_3_ is reported to be more effective than treatment with CN-A or vitamin D_3_ alone in inducing the differentiation of acute monocytic leukemia cells [42]. The effect of CN-A appears to involve a more diverse array of factors compared to other differentiation-inducing reagents. These previous reports have indicated that the effects of CN-A in retinoblastoma cell lines can be extrapolated to other cytokines or differentiation-inducing regents, including RA, butyrate, and dibutyryl cyclic AMP.

In summary, we found that CN-A is a potent inhibitor of cell proliferation and apoptosis, and it changes tumor characteristics in retinoblastoma cell lines. Differences in the effect of CN-A on ocular retinoblastoma tumors and metastatic tumors were observed. CN-A may be useful in researching *PAX6* splice variants. The effect of CN-A on retinoblastoma cells indicates that CN-A may have distinct functions beyond what we have reported here. Further work is required to elucidate the mechanisms underlying these novel observations. This research was supported by a Grant-in-Aid from the Ministry of Education, Culture, Sports, Science, and Technology of Japan.
